# Strengthening of a Near β-Ti Alloy through β Grain Refinement and Stress-Induced α Precipitation

**DOI:** 10.3390/ma13194255

**Published:** 2020-09-24

**Authors:** Wei Chen, Chao Li, Kangtun Feng, Yongcheng Lin, Xiaoyong Zhang, Chao Chen, Kechao Zhou

**Affiliations:** 1 State Key Laboratory of Powder Metallurgy, Central South University, Lu Mountain South Road, Changsha 410083, China; 143111047@csu.edu.cn (W.C.); pkhqchenchao@126.com (C.C.); zhoukechao@csu.edu.cn (K.Z.); 2 Hunan Goldsky Titanium Industry Technology Co., Ltd., No. 97, Qianming Road, Deshan Town, Changde ECO-TECH Development Zone, Changsha 410205, China; jacky19851108@163.com; 3 AVIC Landing Gear Manufacturing Corporation, Changsha 410083, China; fengchao200@sina.com; 4 School of Mechanical and Electrical Engineering, Central South University, Changsha 410083, China; yclin@csu.edu.cn

**Keywords:** near β-Ti alloy, cross-rolled, thermo-mechanical process, microstructure evolution, strengthening mechanism

## Abstract

Near β-Ti alloys with high strength and good ductility are desirable for application in aviation and aerospace industries. Nevertheless, strength and ductility are usually mutually exclusive in structural materials. Here we report a new thermo-mechanical process, that is, the alloy was cross-rolled in β field then aged at 600 °C for 1 h. By such a process, a high strength (ultimate tensile strength: 1480 MPa) and acceptable ductility (elongation: 10%) can be simultaneously achieved in the near β-Ti alloy, based on the microscale β matrix and nanoscale α phase. The microstructure evolution, mechanical properties and strengthening mechanisms have been clarified by scanning electron microscopy (SEM) and transmission electron microscopy (TEM). The results showed that the grain size of the β phase progressively decreased with the increasing of rolling reduction. Moreover, dense dislocation structures and martensite phases distributed in the cross-rolled β matrix can effectively promote the precipitation of nanoscale α particles. TEM analyses confirmed that a heat-treatment twin was generated in the newly formed α lath during aging. These findings provide insights towards developing Ti alloys with optimized mechanical properties.

## 1. Introduction

Low cost and high performance are the target of the aviation and aerospace industries. Near β-Ti alloys are widely used in these two fields owing to their light weight, higher fracture toughness and excellent balance of strength and ductility [1,2,3]. However, it is a complicated task to design an effective processing technology to manufacture the near β-Ti alloys with desirable microstructures which have permitted numerous balances of properties. The abundant microstructures, such as duplex, Widmanstätten, basketweave and equiaxed microstructures, are regulated by the transformation from β-Ti (BCC) to α-Ti (HCP), which are strongly dependent on thermomechanical processes (TMP) [4,5,6].

During TMP, several deformation mechanisms, such as dislocation slip, twinning and stress-induced martensite (SIM) transformation, are well established phenomena in metastable Ti-alloys, depending upon β phase stability [7,8,9,10]. Considerable attention has been focused on the activation of different mechanisms during the plastic deformation in the Ti-alloys with BCC structure. For example, Ti-alloys containing hybrid twinning-induced plasticity (TRIP) and transformation-induced plasticity (TWIP) effects may exhibit an excellent combination of high strength and large ductility [11,12,13]. Besides, pre-existing nanoscale martensite phases can serve as nucleation sites and assist the nucleation and growth of α precipitates (α variants) during subsequent heat treatment (aging or annealing) [14,15,16,17,18,19,20]. Variants with a lamellar or acicular morphology observed in β matrix adopt Burgers orientation relationships (BORs): ${\left\{110\right\}}_{\beta }//{\left\{0001\right\}}_{\alpha }$ and ${\langle 111\rangle }_{\beta }//{\langle 11\bar{2}0\rangle }_{\alpha }$ [21]. Twelve orientational variants with respect to β phase can be equiprobably formed in the β matrix during the phase transition of β → α [22,23,24], which significantly affects the mechanical properties of the Ti-alloys. The displacive phase transformation results in enhanced texture intensities of two main (90°, 90°, 0°) and (90°, 30°, 0°) orientation components [25,26]. By properly tailoring the α + β microstructures, the strength can be significantly enhanced to a relatively high value; however, it also results in a poor ductility.

Submicrometer- or nano-scale ultrafine grained (UFG) materials have been investigated extensively in recent years owing to their new physical properties and excellent mechancial properties [27,28]. Various severe plastic deformation (SPD) techniques have been applied to acquire UFG microstructures, such as equal-channel angular pressing (ECAP) [29,30] and high-pressure torsion (HPT) [31,32]. However, considering the high deformation resistance during the deformation process and heterogeneous deformation domain (such as shear bands [33]), these SPD approaches are hardly applied in near β-Ti alloys. Our previous work [33,34] demonstrated that multi-pass cross-rolling can be employed to refine both of the α phase and β matrix. Meanwhile, lattice distortion remained, and TWIP and TRIP were simultaneously activated in the cross-rolled β matrix. However, the aforementioned process still encounters low efficiency in grain refinement.

Herein, in consideration of abovementioned factors, a novel TMP has been developed in the Ti-55511 alloy. The alloy was firstly cross rolled in β field, then followed by aging at 600 °C for 1 h. By such process, a high strength and acceptable ductility can be simultaneously obtained, which is attributed to microscale β matrix and nanoscale α precipitates. In the present study, the microstructure evolution was characterized. The effects of these factors on the precipitation of the α phase were analyzed. The achieved mechanical properties were also discussed. The acquired results are paving a promising way for designing UFG structures with good balance of strength and ductility.

## 2. Materials and Methods

**Figure 1:**
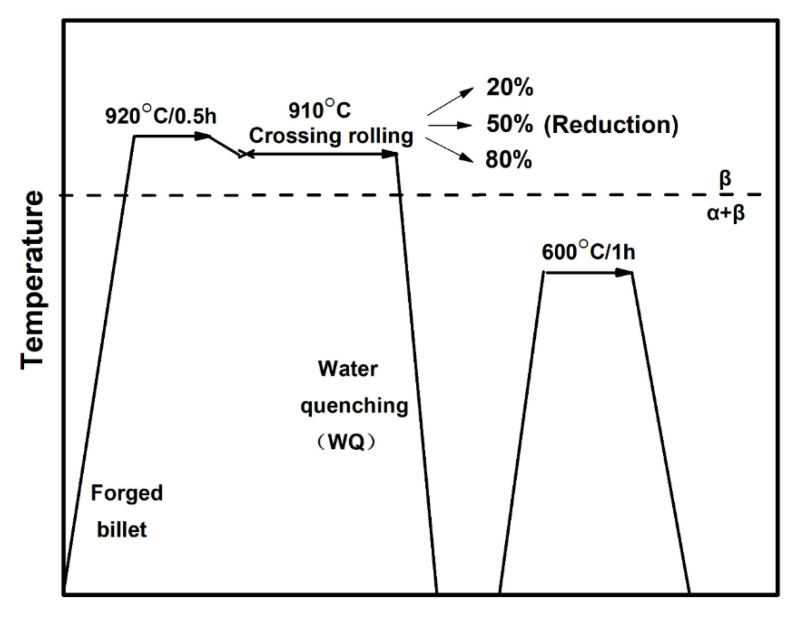
Schematic illustration of processing routes.

The chemical composition of a forged-bar Ti-55511 alloy provided by Xiangtou Goldsky Titanium Industry Co., Ltd. (Changsha, Hunan, China) was used in present study, and it has the following chemical composition (wt.%): 5.16 Al, 4.92 Mo, 4.96 V, 1.10 Cr, 0.98 Fe, and Ti (balance). The β → α transus temperature is approximately measured as 875 ± 5 °C via metallographic observations. Four cuboid samples (150 mm × 45 mm × 15 mm) cut from the forged bar were subjected to the TMP schedule as shown in Figure 1. These cuboid samples were first solution-treated at 920 °C for 0.5 h to acquire the full β microstructure. Then, three of them were cross-rolled in β field with total thickness reductions 20%, 50% and 80%, respectively, followed by quenching in ice water. During cross rolling, 10% reduction was performed in every pass, and the samples were again placed into the furnace to retain the rolling temperature after each pass, and the dwell time was 3 min. Finally, the solution-treated sample and cross-rolled samples were subjected to thermal treatment at 600 °C for 1 h in order to promote the precipitation of α phases.

Tensile specimens were machined out from each rolled sheet with gauge dimensions of 25 mm × 6 mm × 1 mm. Tensile testing was carried out ambient condition using an Instron 3369 mechanical testing machine (Instron, Boston, MA, USA), and every test was repeated five times. The elongation was determined according to displacement of crosshead.

For microstructural characterization and texture analysis by optical microscope (OM, OLYMPUS, Tokyo, Japan), scanning electron microscopy (SEM, Helios Nanolab G3 UC dual-beam microscope system, FEI, Hillsboro, OR, USA) and transmission electron microscopy (TEM; titan G2 60-300, FEI, Hillsboro, OR, USA). Quantitative measurements of grain size were carried out via the Image-Pro-Plus analysis software (Media Cybernetics, MD, USA). The average grain size of β matrix was determined by IPP (Image-Pro Plus) software.

The TEM specimen was mechanically ground into thin foils (40–50 μm) and electropolished (jet thinning) with Kroll’s reagent (ASTM 192), which consists of 60% methanol, 35% butanol, and 5% perchloric acid, using a twin-jet electropolishing device (Denmar Struers A/S, Ballerup, Danmark).

## 3. Results

### 3.1. Refinement of β Grains during Cross Rolling

**Figure 2:**
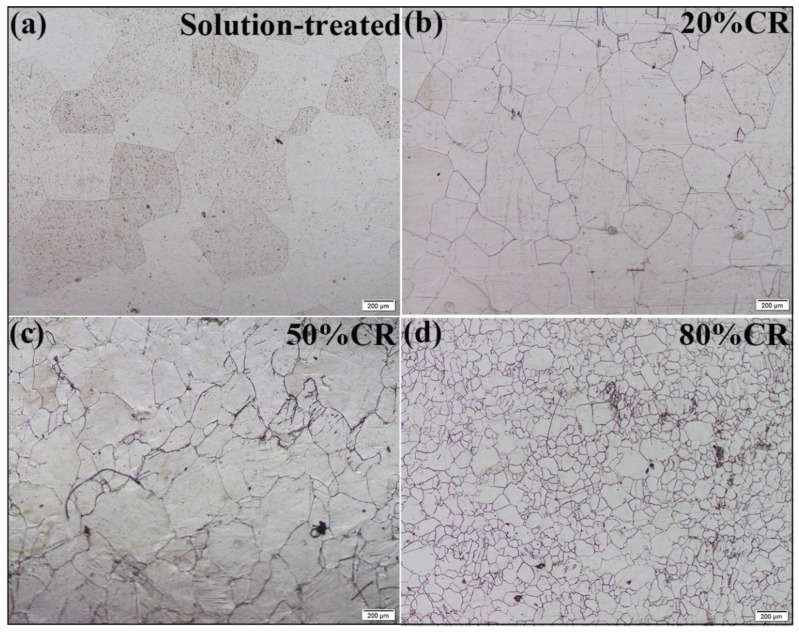
Optical micrographs of (**a**) the solution-treated sample, and (**b**–**d**) the cross-rolled samples with rolling reductions of 20%, 50% and 80%, respectively.

The rolling microstructures were examined by optical microscopy (OM). In the un-deformed state (solution-treated), the sample has a relatively coarse prior β grains (Figure 2a). Figure 2b–d show the morphologies of the cross-rolled samples with thickness reductions of 20%, 50% and 80%, respectively. It can be seen that the increasing rolling reduction promotes the refinement of β grains, from 609 μm (solution-treated) to 141 μm (80% reduction). When the reduction reaches 80%, the smallest size of β grain can be obtained. This indicates that the cross rolling can effectively refine the β grain size. The previous studies [33,34] demonstrate that cross rolling can induce more deformation than unidirectional rolling and accelerate the activation of dislocation slip, which can effectively trigger the formation of β subgrains and promote dynamic recrystallization (DRX) β grains. Typical microstructures of the sample with 80% rolling reduction was chosen to be investigated as follows.

### 3.2. Martensite Phases and Dislocation Substructures in the Cross-Rolled β Matrix

**Figure 3:**
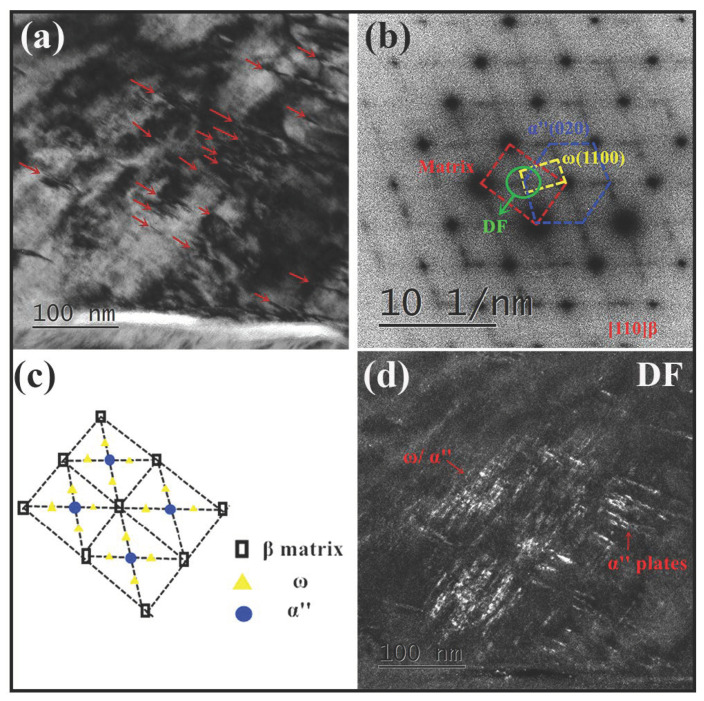
(**a**) BF-TEM image of α” phases (indicated by the red arrows) observed in the 80% cross-rolled sample; (**b**) SAED pattern along ${\left[110\right]}_{\beta }$ zone axis corresponding to Figure 3a; (**c**) Schematic illustrating the position of diffraction spots in Figure 3b (black: β spots; yellow: ω spots, blue: α” spots); (**d**) DF-TEM micrograph taken from the diffraction spots indicated by the green circle in Figure 3b.

**Figure 4:**
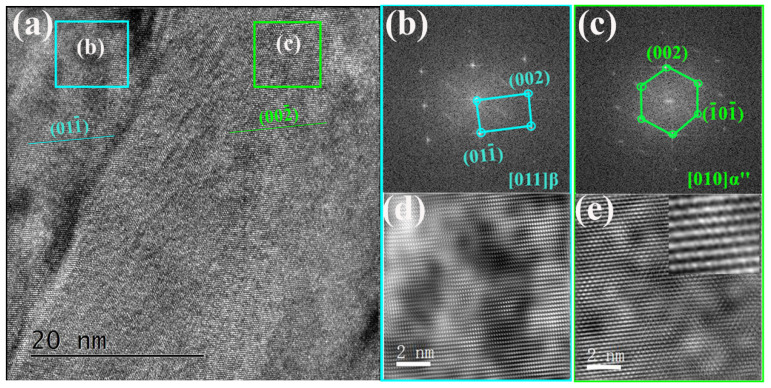
(**a**) HRTEM image of the α” phase; (**b**,**c**) the FFT patterns of areas b and c in Figure 4a; (**d**,**e**) the corresponding Fourier filtered HRTEM images of areas b and c.

TEM analyses of the 80% cross-rolled sample display details of transformation products (Figure 3). The bright-field (BF) TEM image shows parallel and nano-sized lamellae within β matrix (marked by red arrows), as seen in Figure 4a. In view of the solution-treated condition, two kinds of phases showing in the corresponding selected area electron diffraction (SAED) pattern along ${\left[110\right]}_{\beta }$ zone axis (Figure 3b) are highlighted in blue (α” phase) and yellow color (ω phase), respectively, and the diffraction spots of these two martensite phases are schematically illustrated in Figure 3c. Selecting the diffraction spots (green circle) corresponding to α” and ω phases, the dark-field (DF) image of Figure 3d shows a primary microstructure of zigzag-shaped α” plates as well as a small amount of ω phases (irregular shape). The ω phase is concentrated near the α” phase, as shown in Figure 3d. The acquired martensite phases formed by deformation process (SIM transformation) and also by quenching process [15,35,36].

The atomistic structure of the α”phase within β matrix was further analyzed by high-resolution TEM (HRTEM) under the zone axis of ${\left[110\right]}_{\beta }$ (Figure 4). The orientation relationships (OR) between the β matrix (area b) and α” phase (area c) in Figure 4a were verified by the inserted fast Fourier transformation (FFT) patterns. As presented in Figure 4b,c, the OR between β matrix and α” phase is $<011>\beta //<010>\alpha^{\prime \prime }$. The difference between the crystal structure of the β matrix and α” phase was revealed by the corresponding Fourier filtered HRTEM images, as presented in Figure 4d,e. Based on the schematic crystal structures (Figure 3c) and FFT patterns, the lattice parameters of the β phase were measured to be a = 0.295 nm; and the α” phase were measured to be a = 0.260 nm, b = 0.443 nm and c = 0.457 nm.

**Figure 5:**
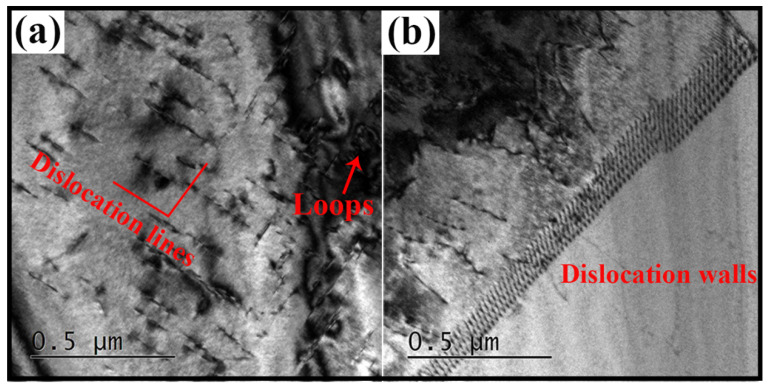
TEM micrographs of the 80% cross-rolled sample showing the formation of (**a**) dislocation lines and loops, and (**b**) dislocation walls in β matrix.

The dislocation configuration of the 80% cross-rolled sample was examined by TEM (Figure 5). A large number of dislocation structures, such as perpendicular and straight dislocation lines, loops (Figure 5a) and walls (Figure 5b) were observed in β field. The observed results showed that dislocation slip dominates plastic deformation in soft β matrix with BCC structure.

### 3.3. Microstructure after Aging

It is well accepted that the α phase is the most common strengthening phase for near-β alloys. High strength of the two-phase alloy is mainly related to the precipitation hardening effect during the heat treatment process. In comparison with the equiaxed microstructure, the lamellar form shows higher tensile strength. However, generally, with the increasing amount of the α phase, the ductility of the alloy will decrease sharply. Therefore, one of the most direct methods to improve strength and ductility simultaneously is to produce an UFG microstructure. The development of UFG microstructures is generally along two distinctive directions: breaking down the pre-existing microstructure by severe plastic deformation (SPD) [30,31,32,37,38], and/or introducing nano-scaled precipitates by heterogeneous nucleation [14]. The previous one has successfully been applied in many HCP materials. However, owing to high deformation resistance, the near β-Ti alloy is not a suitable candidate for SPD processes in consideration of the feasibility and economic issues. The latter one is mainly reported in metastable β-Ti alloys, in which the nano-scaled α phase precipitates from the heterogeneous nucleation sites, such as martensite phases (ω, α’ and α”) from deformation and heating processes [14]. Besides, the dislocation structure can also be considered as the precursor to α precipitations [39,40].

**Figure 6:**
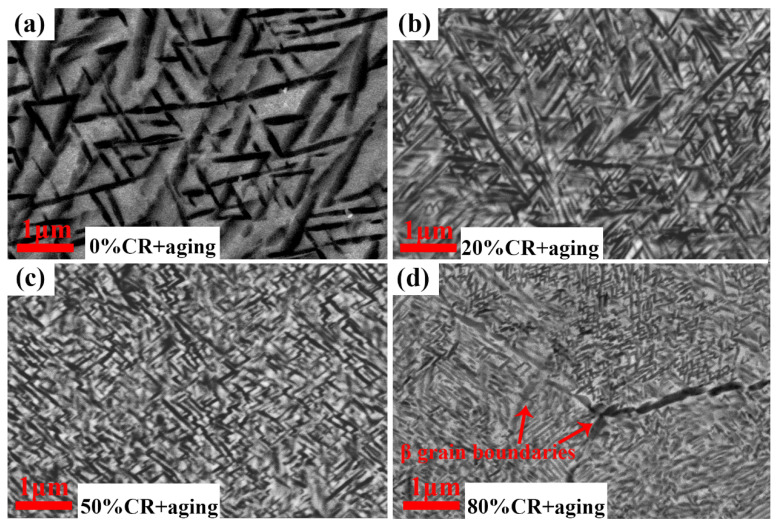
SEM images of the solution-treated sample and cross-rolled samples aged at 600 °C for 1 h. (**a**) 0% + aging, (**b**) 20% + aging, (**c**) 50% + aging, (**d**) 80% + aging.

To rationalize this fact, the solution-treated sample and the cross-rolled samples with different rolling reductions were then aged at 600 °C for 1 h (named: sample 0% CR + aging, 20% CR + aging, 50% CR + aging and 80% CR + aging, respectively), and the morphologies of these samples are presented in Figure 6. Figure 6a shows a typical triangular distribution within the β matrix of sample 0% CR + aging, which is attributed to selected α phase variants precipitating from the parent β phase with BORs. From Figure 6a–c, it can be seen that the grain size of α phase decreased with the increasing of rolling reduction. Besides, the grain sizes of the β grain in sample 80% CR + aging are significantly smaller than sample 50% CR + aging.

**Figure 7:**
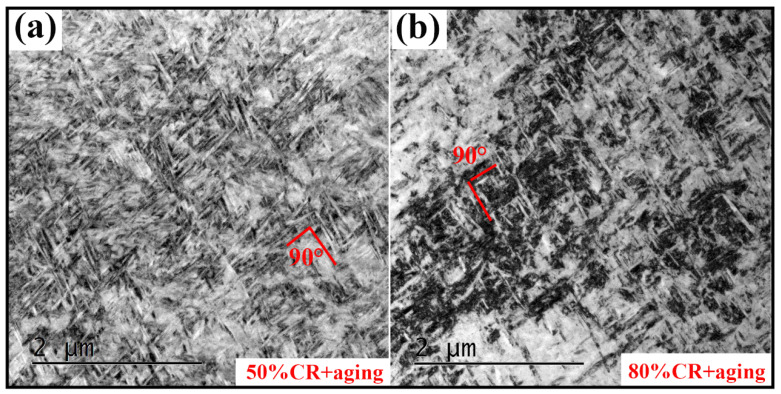
High-magnification TEM images of samples (**a**) 50% + aging and (**b**) 80% + aging.

The characteristics of α grains in samples 50% CR + aging and 80% CR + aging were further characterized by TEM analysis. Figure 7 shows the high-magnification TEM images of samples 50% CR + aging and 80% CR + aging. As depicted in Figure 6, the grain sizes of these two samples (50% CR + aging and 80% CR + aging) are obviously smaller than those of samples solution-treated and 20% + aging. The formation of uniform and refined α precipitates mainly relies on pre-existing precursors, such as martensite phases and dislocation structures, which can provide a strong driving force of the nucleation and growth of the α phase [39]. Besides, the BF images of these two samples with refined microstructures exhibits some fine distribution of the variant of α laths which are oriented 90°, as shown in Figure 7a,b, while a triangularly distributed one is more obvious in the non-deformed sample (Figure 6a). In general, twelve α variants with BORs can be equiprobably generated in a parent β grain, and they can be combined with each other to form six orientations. The preferred orientation might be ascribed to the special dislocation array, as depicted in Figure 5. Previous work [24,40,41] demonstrated that the dislocation structure plays an in the variant selection of α phase during the β→α phase transformation. For example, the habit plane orientation of α phase tend to parallel and/or perpendicular to the dislocation lines to yield a large form.

### 3.4. Mechanical Properties

**Figure 8:**
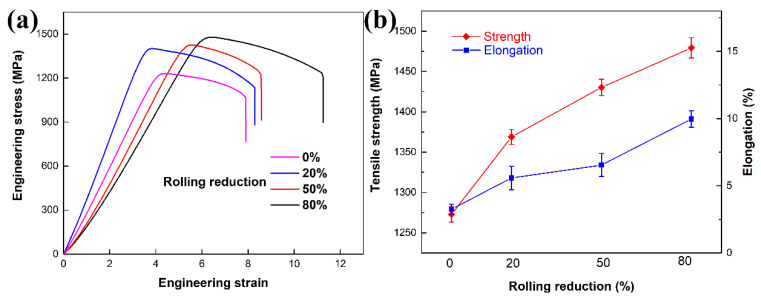
(**a**) Engineering strain-stress curves the aged samples. (**b**) Variations of average the ultimate tensile strength and elongation with rolling reduction.

The tensile results are shown in Figure 8a, with Figure 8b showing the variations of average ultimate tensile strength and elongation with rolling reduction. It is obvious that the ultimate tensile strength and elongation increased with increasing rolling reduction. Sample 80% CR+ aging exhibits the highest ultimate tensile strength of 1480 MPa among all the samples, which is approximately 16% higher than that of the sample with the normal lamellar microstructure (sample 0% CR + aging). It was reported that, as the strength higher than 1200 MPa, the elongation is usually lower than 10% (even lower than 5%) in the lamellar near β-Ti alloy without α + β filed deformation [3,42,43,44]. However, the highest elongation of 10.1% was achieved in the sample with the highest ultimate tensile strength as well. It is thus surprising to observe the simultaneous enhancement in strength and ductility in the pre-cross-rolled samples.

There are two main factors contributing to this phenomenon. On the one hand, nanoscale α precipitates and microscale β grains were wildly distributed in the pre-deformed samples, which results in the increase of tensile strength. On the other hand, the grain size of β-grain decreased with increasing rolling reduction. The formation of refined β phases can effectively prohibit crack propagation and hence improve the ductility of alloys [45].

### 3.5. Fracture Morphologies

**Figure 9:**
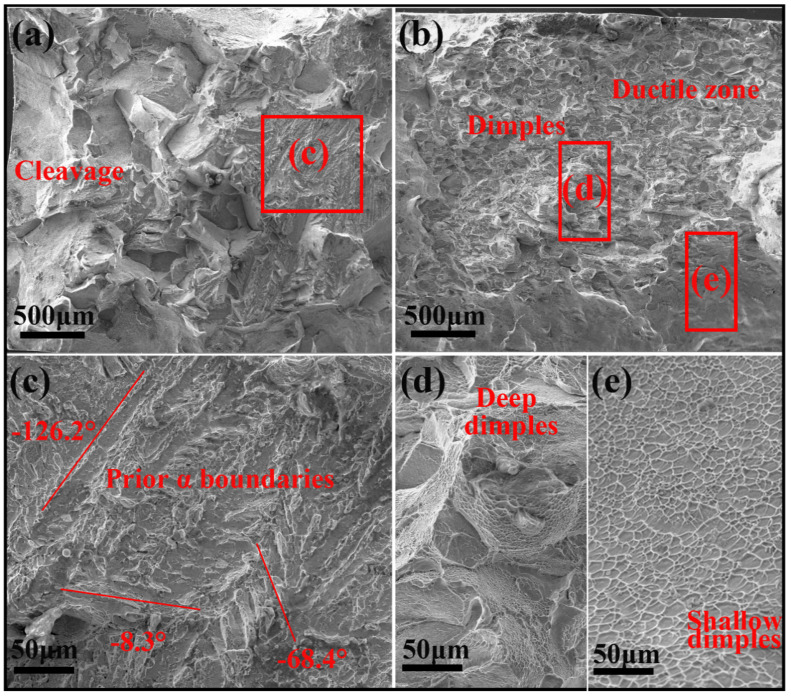
Fractographs of (**a**) sample 0% + aging and (**b**) sample 80% + aging; high-magnification images of area c in Figure 9a (**c**), and areas d and e in Figure 9b (**d**,**e**), respectively.

Fracture morphologies were characterized in Figure 9. Figure 9a,b show the tensile fracture surfaces of samples 0% + aging and 80% + aging, respectively. The fracture morphology of sample 0% + aging shows a dominant brittle fracture, mainly composed by continuous cleavages and tearing ridges (Figure 9a). A lot of tearing ridges can be observed at the high magnification image of the area marked in Figure 9a (Figure 9c). The tearing ridges might be attributed to the crack initiation and propagation along the α laths, because the extension directions of these ridges (marked by red lines) accord well with the prior triangular-distributed α phase precipitating from the parent β phase with BORs (Figure 6a). In contrast, deep (Figure 9d) and shallow (Figure 9e) dimples were abundant in the fracture surface of sample 80% + aging, which shows a characteristic of ductile fracture.

**Figure 10:**
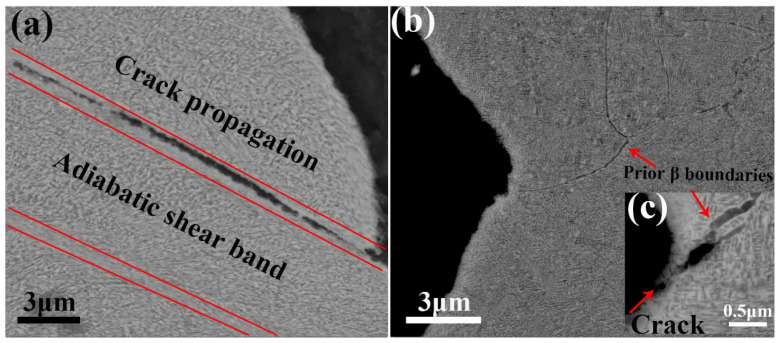
Profile-view SEM micrographs closed to the fracture surface of samples (**a**) 0% CR+ aging and (**b**) 80% CR+ aging. (**c**) An enlarged image showing a micro-crack along the grain boundary.

To clarify failure mechanisms during dynamic loading, the profile-view SEM images close to the fracture surface of these two samples were further analyzed, as shown in Figure 10. Two long and narrow adiabatic shear bands (ABS) are noted in sample 0% CR+ aging (Figure 10a), which are distinct from the matrix. Moreover, a long crack propagated along one of the bands. It means adiabatic shear failure occurs in sample 0% CR+ aging during dynamic loading. ABS, as an unstable deformation mode, unually developes in Ti and Ti-alloys during high strain-rate or/and high temperature deformation due to their low heat conductivity and high adiabatic shearing sensitivity. Whereas, the similar morphology was not observed in the surface of sample 80% CR+ aging (Figure 10b). Meanwhile, a micro-crack is clearly observed in a high-magnification image (Figure 10c), and it is also clear that the crack path was blocked at the β grain boundary, which is accordance with the higher ductility of sample 80% CR+ aging.

### 3.6. Heat-Treatment $\left\{10\bar{1}1\right\}$ Twinning

Twinning has been well investigated and applied recently in numerous metals due to their strengthening effect via subdividing grains (Hall–Petch relation). The strengthening effect comes from the fact that the twin boundaries block the lattice dislocation motion [46]. In HCP and low symmetry metals and alloys, such as Ti, Mg and their alloys, twins are usually activated during deformation, owing to their insufficient number of slip systems. In our previous work [33,34,47], the $\left\{10\bar{1}1\right\}$-type twinning has been systematically characterized in hot-deformed near β-Ti alloys. However, to the authors’ knowledge, $\left\{10\bar{1}1\right\}$ twins has never been observed during heat treatment.

**Figure 11:**
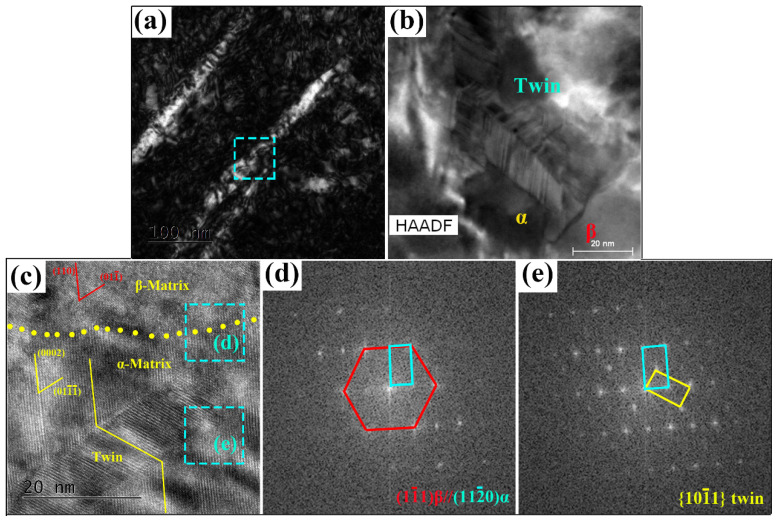
(**a**) TEM image of the sample 80% + aging. (**b**) Aberration-corrected HAADF images and (**c**) HRTEM image showing the formation of α-twin bands in the α lath. The corresponding FFT patterns revealing: (**d**) the BOR between the α and β phases, and (**e**) a $\left\{10\bar{1}1\right\}$- type nano-twin observed in the α lath.

With a much higher resolution, HRTEM and aberration-corrected high-resolution HAADF were applied to examine the microstructure in more detail of sample 80% + aging. As shown in Figure 11a,b, parallel α laths are observed in the β matrix, and band structures show in the α lath. HRTEM imaging was employed to view the atomistic structure of one α lath. Figure 11c presents an HRTEM image of the α lath under the zone axis of ${\left[\bar{1}11\right]}_{\beta }$. A long and regular-shaped band shows in the center of the α lath. The ORs between α and β matrix (framed area d), and the long band and α matrix (framed area e) were further verified by the FFT pattern, as seen in Figure 11d and e, respectively. According to the FFT pattern shown in Figure 11d,e, the OR between α and β matrix obey BOR: ${\left(1\bar{1}1\right)}_{\beta }//{\left(11\bar{2}0\right)}_{\alpha }$, and the long band is $\left\{10\bar{1}1\right\}$-type twin. Generally, no matter the lamellar morphology (in Figure 11a) or BOR, both of them reveal that the α phase formed by the cooling from the β field without plastic deformation [48]. Therefore, it is surprising that the activation of the $\left\{10\bar{1}1\right\}$ twin within the lamellar α phase.

It is generally understood that the $\left\{10\bar{1}1\right\}$ twinning is required to accommodate plastic deformation along the c-axis for near β-Ti alloys [33,46]. As shown in Figure 11c, the matrix (α or β) around the α/β twin boundary is distorded compared with the other regions, which implies a high stress concentration at the α/β twin boundary. The stress concentration in β matrix results from the cross-rolling process, and hence, the activation of twins in the lamellar α phase was proposed could accommodate local stress during α growth. Moreover, it was reported that the mechanical twinning is usually accompanlied with SIM transforamtion [49,50]. In present research, the SIM transformation (martensite phases→α phase) plays a critical role in the formation of α laths during aging.

- type nano-twin observed in the α lath.

## 4. Conclusions

In summary, the Ti-55511 alloy was subjected to a new thermo-mechanical, that is, the alloys were firstly cross-rolled in β field, and then aged at 600 °C for 1 h. The evolution of microstructures, mechanical properties and strengthening mechanisms were systematically investigated. The detailed features are summarized as follows:

(1) The cross-rolling method has a great influence on the grain refinement of the β phase. Meanwhile, nano-sized martensite phases of α” + ω and dense dislocation structures, e.g., dislocation lines, loops and tangles, were introduced into the cross-rolled β matrix.
(2) The pre-existing nano-sized martensite phases and dense dislocation structures can promote the precipitation of UFG α phases during subsequent aging. Moreover, the grain size of the α phase was dependent on the rolling reduction. The β grain size decreased with the increasing rolling reduction.
(3) By this process, a higher level of strength (1480 MPa) can be achieved, and still preserving an acceptable ductility (10%) in this alloy. The high strength mainly results from the refinement of prior β grains and nano-sized α precipitate and the enhanced ductility is strongly dependent upon the β grain size.
(4) Furthermore, $\left\{10\bar{1}1\right\}$ twins formed inside the α lath during the heat treatment. The activation of twins in α phases could accommodate local stress during α growth. Considering that the strengthening mechanism of α-twinning, this work can be pursued in future investigations by multiscale studies.
